# Supplementary material for: Biosynthetic Melanin/Ce6-Based Photothermal and Sonodynamic Therapies Significantly Improved the Anti-Tumor Efficacy
Source: Pharmaceutics. 2023 Jul 31;15(8):2058. doi: 10.3390/pharmaceutics15082058 (PMC10457960; doi:10.3390/pharmaceutics15082058)
Supplement: Supplementary file 1 [file pharmaceutics-15-02058-s001.zip › pharmaceutics-2473052-supplementary.pdf]

pharmaceutics

MDPI

Supplementary Materials:

Biosynthetic melanin/Ce6-based photothermal and sonodynamic therapies significantly improved the anti-tumor efficacy

Yuping Yang [1,2,3], Yaling He [4] , Meijun Fu [4], Meijun Zhou [2], Xinxin Li [4], Hongmei Liu [1,2,*] and Fei Yan [4,*]

ATGGGCAGCAGCCATCACCATCATCACCACAGCCAGGATCCGGCGCGCCATGGG-
TAACAAGTATAGAGTTAGAAAAAACGTATTACATCTTACCGACACGGAAAAAAGAGATTTT-
GTTCGTACCGTGCTAATACTAAAGGAAAAAGGGATATATGACCGCTATATAGCCTGG-
CATGGTGCAGCAGGTAAATTTCATACTCCTCCGGGCAGCGATCGAAATGCAGCACATATGAG-
TTCTGCTTTTTTACCGTGGCATCGTGAATACCTTTTACGATTCGAACGTGACCTTCAG-
TCAATCAATCCAGAAGTAACCCTTCCTTATTGGGAATGGGAAACGGACGCACAGATGCAG-
GATCCCTCACAATCACAAATTTGGAGTGCAGATTTTATGGGAGGAAACGGAAATCCCATAAAA-
GATTTTATCGTCGATACCGGGCCATTTGCAGCTGGGCGCTGGACGAC-
GATCGATGAACAAGGAAATCCTTCCGGAGGGCTAAAACGTAATTTTGGAGCAACGAAA-
GAGGCACCTACACTCCCTACTCGAGATGATGTCCTCAATGCTTTAAAAATAACTCAGTATGA-
TACGCCGCCTTGGGATATGACCAGCCAAAACAGCTTTCGTAATCAGCTTGAAGGAT-
TTATTAACGGGCCACAGCTTCACAATCGCGTACACCGTTGGGTTGGCGGACAGATGGGCGTT-
GTGCCTACTGCTCCGAATGATCCTGTCTTCTTTTTACACCACGCAAATGTGGATCGTATTT-
GGGCTGTATGGCAAATTATTCATCGTAATCAAAACTATCAGCCGATGAAAAACGGGCCATTT-
GGTCAAAACTTTAGAGATCCGATGTACCCTTGGAATACAACCCCTGAAGACGTTATGAAC-
CATCGAAAGCTTGGGTACGTATACGATATAGAATTAAGAAAATCAAAACGTTCCTCATAA

Figure S1. The open reading frame sequence of tyrosinase gene from B. megaterium.

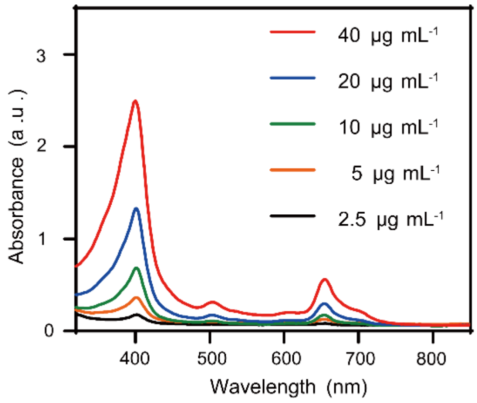

Figure S2. The photo absorbance spectra of Ce6 at varied concentrations.

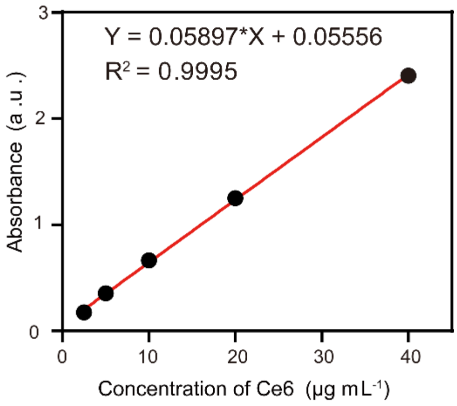

Figure S3. The concentration-dependent absorbance of Ce6 at the wavelength 404 nm.

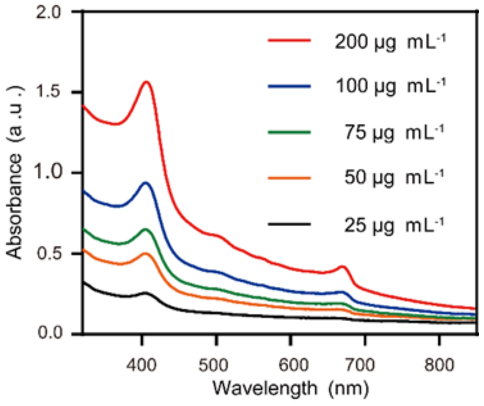

Figure S4. The photo absorbance spectra of MC@Lip at different concentrations.

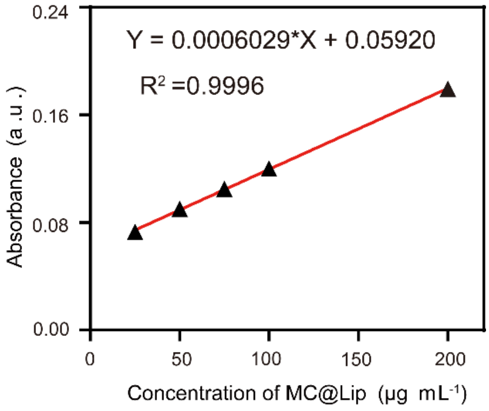

Figure S5. The concentration-dependent absorbance of MC@Lip at the wavelength 808 nm.

And the η value which is the photothermal conversion efficiency was calculated as follows:

$$\eta_T = \frac{hA(\Delta T_{\max} - \Delta T_{\max,H2O})}{I(1-10^{-A_{808}})} \quad \tau_s = \frac{m_{H2O} c_{H2O}}{hA}$$

ΔTmax and ΔTmax,H2O stand for the temperature elevation of MC@Lip and deionized water under the irradiation of 808 nm laser for 5 min. The I is the laser power, and A808 is the absorbance of MC@Lip. The h and A represent the heat transfer coefficient and surface area of container. mH_2O and cH_2O are the mass (0.2 g) the heat capacity (4.2 J g^{-1}) of deionized water.

ΔT	A_{808}	I（W）	τ_s	h_A	η
20.0	0.1795	1.00	116.8	0.00719	42.47 %

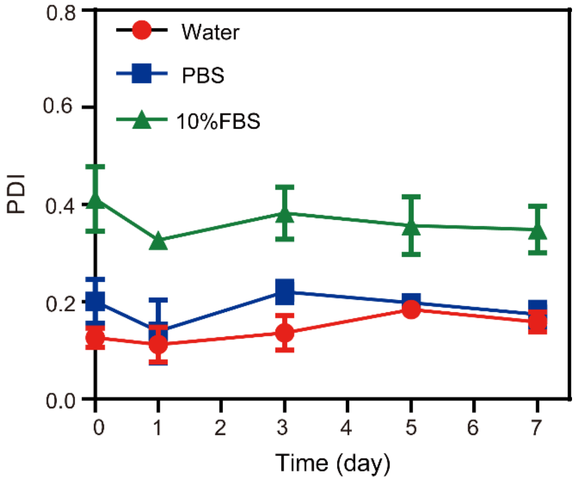

Figure S6. Changes in the polydispersity indexes of MC@Lip stored in water, PBS or 10% FBS at 4°C for different durations.

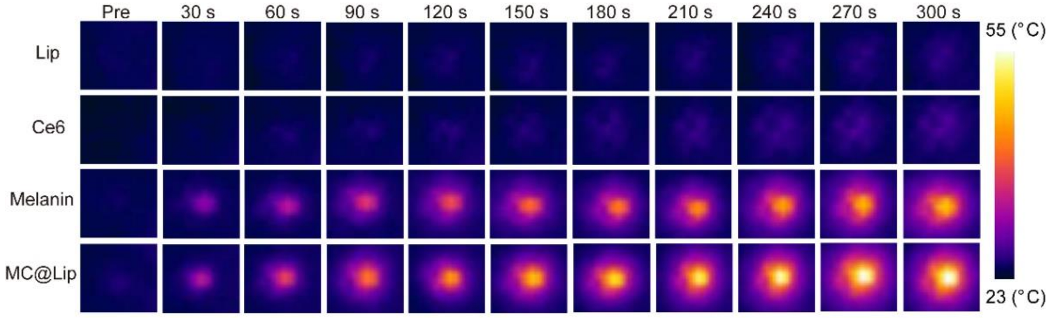

Figure S7. IR thermal images of MC@Lip (melanin concentration: 200 μg mL^{-1}), Lip, melanin (200 μg mL^{-1}) and Ce6 (12 μg mL^{-1}) suspension under 5 min NIR irradiation (808nm, 1.00 W cm^{-2}).

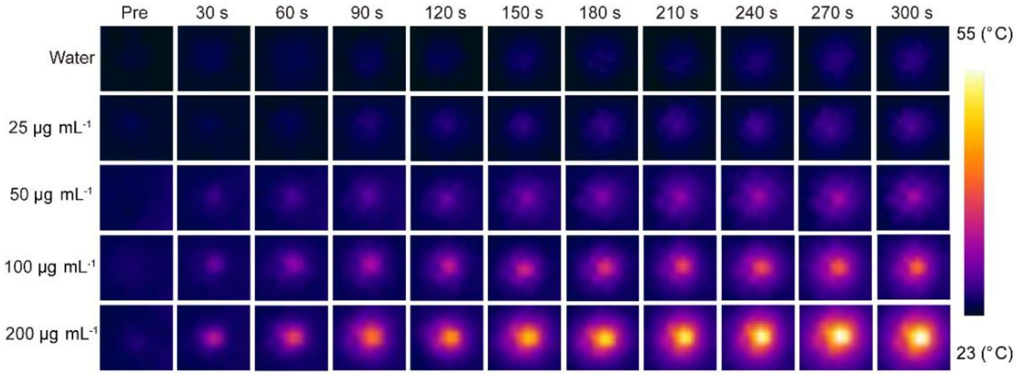

Figure S8. IR thermal images of pure water and MC@Lip suspension at different concentrations (25, 50, 100 and 200 μg mL^{-1}) under 5 min NIR irradiation (808nm, 1.00 W cm^{-2}).

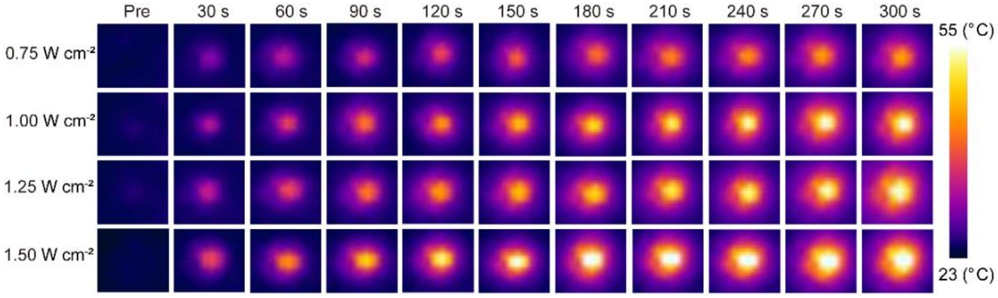

Figure S9. IR thermal images of MC@Lip (melanin concentration: 200 μg mL^{-1}) suspension under different power densities of 5 min NIR irradiation (808 nm, 0.75, 1.00, 1.25 and 1.50 W cm^{-2}).

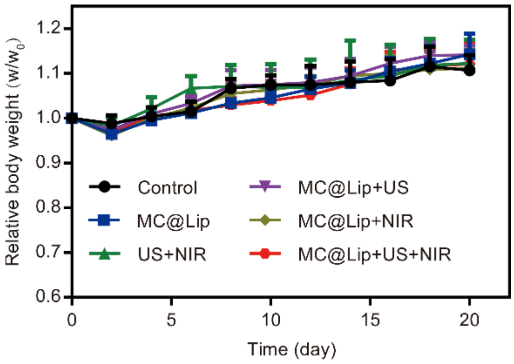

Figure S10. Mice weight curves (n = 5) of six groups after various treatments.

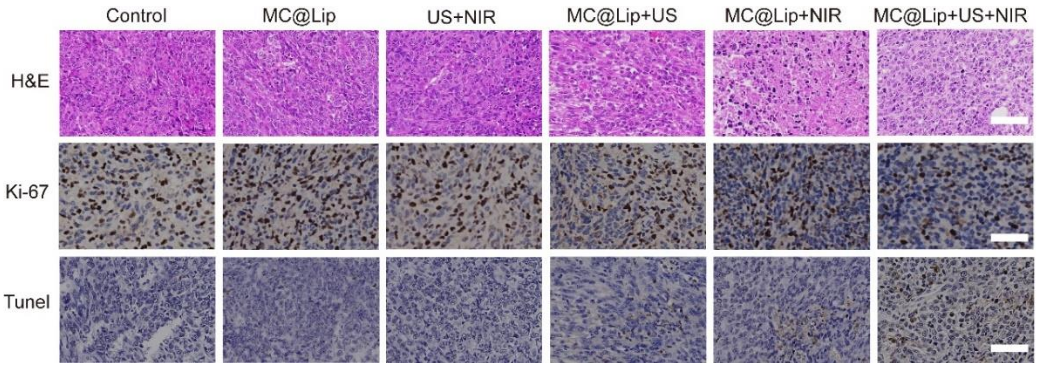

Figure S11. H&E staining, immunochemical staining of Ki-67 and TUNEL staining in tumor region of six groups after different treatments. Scale bar = 50 μm.

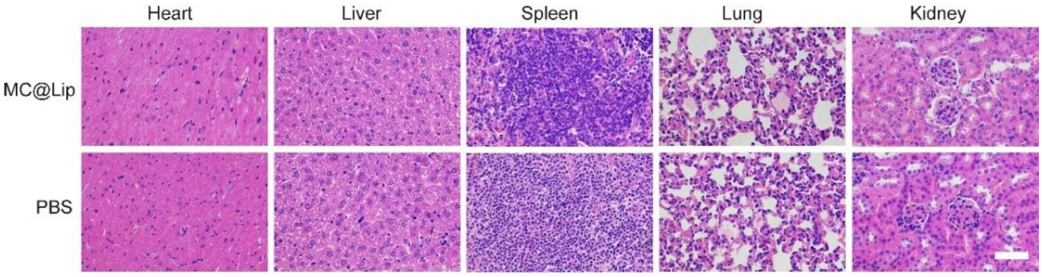

Figure S12. H&E-stained organ slices of healthy mice injected with MC@Lip dispersion or PBS solution after 14 days. Scale bar = 50 μm.

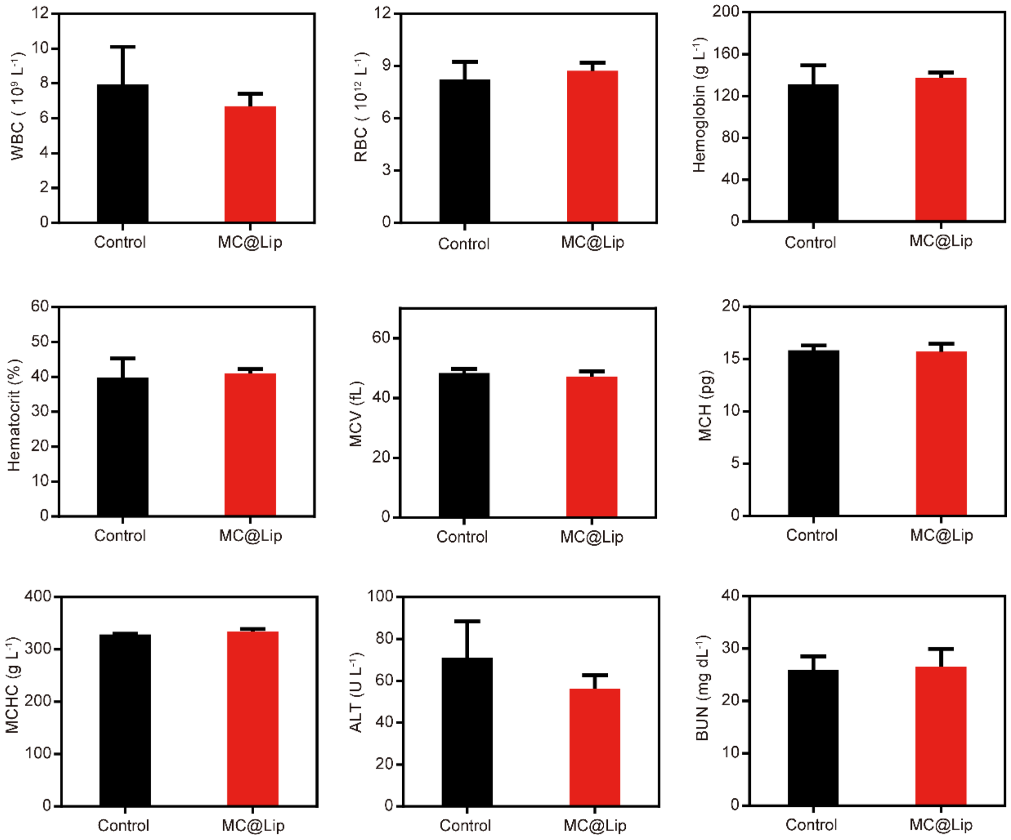

Figure S13. Blood biochemistry and hematology data of healthy mice injected with MC@Lip dispersion or PBS solution after 14 days.
